# Fear of movement was associated with sedentary behaviour 12 months after lumbar fusion surgery in patients with low back pain and degenerative disc disorder

**DOI:** 10.1186/s12891-023-06980-z

**Published:** 2023-11-10

**Authors:** Max Jakobsson, Maria Hagströmer, Hanna Lotzke, Philip von Rosen, Mari Lundberg

**Affiliations:** 1 Division of Physiotherapy, Department of Health and Rehabilitation, Institute of Neuroscience and Physiology, University of Gothenburg, Gothenburg, Sweden; 2 Division of Physiotherapy, Department of Neurobiology, Care Sciences and Society, Karolinska Institutet, Huddinge, Sweden; 3 Academic Primary Health Care Centre, Region Stockholm, Stockholm, Sweden; 4 The Back in Motion Research group, Department of Health Promoting Science, Sophiahemmet University, Box 5605, Stockholm, SE 11486 Sweden; 5 Department of Rehabilitation, Ängelholm Hospital, Ängelholm, Sweden; 6 Sahlgrenska Academy, University of Gothenburg Centre for Person-Centred Care (GPCC), University of Gothenburg, Gothenburg, Sweden

**Keywords:** Accelerometer, Kinesiophobia, Pain catastrophising, Physical activity, Movement behaviour, Predictors, Self-efficacy, Spine Surgery, Postoperative outcomes

## Abstract

**Background:**

Movement behaviours, such as sedentary behaviour (SB) and moderate to vigorous physical activity (MVPA), are linked with multiple aspects of health and can be influenced by various pain-related psychological factors, such as fear of movement, pain catastrophising and self-efficacy for exercise. However, the relationships between these factors and postoperative SB and MVPA remain unclear in patients undergoing surgery for lumbar degenerative conditions. This study aimed to investigate the association between preoperative pain-related psychological factors and postoperative SB and MVPA in patients with low back pain (LBP) and degenerative disc disorder at 6 and 12 months after lumbar fusion surgery.

**Methods:**

Secondary data were collected from 118 patients (63 women and 55 men; mean age 46 years) who underwent lumbar fusion surgery in a randomised controlled trial. SB and MVPA were measured using the triaxial accelerometer ActiGraph GT3X+. Fear of movement, pain catastrophising and self-efficacy for exercise served as predictors. The association between these factors and the relative time spent in SB and MVPA 6 and 12 months after surgery was analysed via linear regression models, adjusting for potential confounders.

**Results:**

Preoperative fear of movement was significantly associated with relative time spent in SB at 6 and 12 months after surgery (β = 0.013, 95% confidence interval = 0.004 to 0.022, p = 0.007). Neither pain catastrophising nor self-efficacy for exercise showed significant associations with relative time spent in SB and MVPA at these time points.

**Conclusions:**

Our study demonstrated that preoperative fear of movement was significantly associated with postoperative SB in patients with LBP and degenerative disc disorder. This finding underscores the potential benefits of preoperative screening for pain-related psychological factors, including fear of movement, preoperatively. Such screenings could aid in identifying patients who might benefit from targeted interventions to promote healthier postoperative movement behaviour and improved health outcomes.

**Supplementary Information:**

The online version contains supplementary material available at 10.1186/s12891-023-06980-z.

## Background

Although there is a growing body of research on fusion surgery for lumbar degenerative conditions [1–8], a significant knowledge gap remains: studies often neglect physical activity as an outcome, despite its associations with crucial health parameters [9–11]. Highlighting its significance, the World Health Organization (WHO) recommends physical activity for all, irrespective of their health condition [12]. Echoing this sentiment, a publication in the Lancet emphasizes the necessity of incorporating physical activity into healthcare strategies for patients with low back pain [13]. In light of this, we previously showed, using objective measures of physical activity, that 83% of the patients with LBP and degenerative disc disorder scheduled for lumbar fusion surgery did not reach the WHO recommendations on physical activity before surgery [14]. Others have found similar results [15]. Patients scheduled for surgery may, therefore, be at risk for poor health due to physical inactivity.

To design prehabilitation and rehabilitation programmes that adequately support patients to be more physically active and less sedentary, it is imperative to move beyond cross-sectional data and instead measure patients’ physical activity both before and after surgery. It is also important to consider physical activity in all its complexity, encompassing a spectrum of behaviours such as sedentary behaviour (SB, e.g. watching television), light intensity physical activity (e.g. doing the dishes), and moderate to vigorous physical activity (MVPA, e.g. brisk walking or jogging) [16]. MVPA and SB, in particular, have been associated with multiple aspects of health [9–11]. For instance, MVPA is strongly related to a reduced risk of chronic disease and premature death [10, 11], and a high amount of SB is associated with an increased risk of premature death [11].

Understanding the complexity of post-surgical movement behaviours necessitates a closer examination of potential barriers to successful outcomes following surgery for lumbar degenerative conditions, such as pain-related psychological factors. Such factors, encompassed in the fear-avoidance model by Vlaeyen et al. [17] and subsequent modifications of that model [18, 19], include fear of movement, pain catastrophising, and poor self-efficacy. In a prior longitudinal study of ours, heightened levels of fear of movement were associated with greater disability after lumbar spine surgery [20]. Moreover, previous research has indicated that elevated preoperative pain catastrophising is associated with poorer postoperative outcomes, including greater pain and disability [21, 22]. Yet, a recent systematic review presented mixed evidence on the associations between pain-related psychological factors and postoperative outcomes of lumbar degenerative conditions [23]. While these studies shed light on the complex interplay of pain-related psychological factors with traditional surgical outcomes, such as pain and disability, the association between pain-related psychological factors and postoperative movement behaviour remains largely unexplored.

In another one of our longitudinal studies, we found that preoperative self-efficacy for exercise was significantly associated with postoperative movement behaviour in terms of MVPA while fear of movement and pain catastrophising were not [24]. However, the follow-up period in that study was only six months. This finding underscores the need for more comprehensive longitudinal research to study the influence of pain-related psychological factors on postoperative movement behaviour.

To our knowledge, no study has yet investigated the association between preoperative pain-related psychological factors on movement behaviour 12 months after lumbar fusion surgery.

## Methods

### Aim

The aim of the study was to evaluate the association between preoperative pain-related psychological factors (fear of movement, pain catastrophising and exercise self-efficacy) and postoperative SB and MVPA in patients with LBP and degenerative disc disorder at 6 and 12 months following lumbar fusion surgery.

### Hypothesis

Preoperative fear of movement, pain catastrophising and self-efficacy for exercise are significantly associated with the relative time spent in SB and MVPA at 6 and 12 months after lumbar fusion surgery for patients with LBP and degenerative disc disorder.

### Design

The study involved secondary analyses of data in a randomised controlled trial studying a person-centred prehabilitation programme [19]. The findings of the primary outcome of that study are published elsewhere [25].

### Characteristics of the population

Patients aged 18–70 years with dominant chronic LBP and degenerative disc disorder affecting 1–3 segments of the lumbar spine scheduled for lumbar fusion surgery were included [19, 25]. Exclusion criteria were spinal malignancy, confirmed neurological or rheumatic disorder, previous decompression surgery for spinal stenosis, deformities in the thoracolumbar spine or poor understanding of the Swedish language.

### Procedure

One hundred and eighteen patients scheduled for lumbar fusion surgery were included from a university hospital and two spine clinics in Gothenburg, Sweden, between 1 April 2014 and 1 July 2017. The patients were randomised to either a person-centred prehabilitation programme or a control group [19, 25]. Measurements were made at 8 to 12 weeks before surgery and again at 6 and 12 months after surgery. During these time points, patients met with an independent observer at one of the spine clinics, completed questionnaires, and received an accelerometer (see below).

### Measurement of movement behaviour

The movement behaviours SB, low-intensity physical activity (LIPA) and MVPA were measured by the triaxial accelerometer ActiGraph GT3X+ (ActiGraph, Pensacola, FL, USA). Procedures for data collection and processing have been reported elsewhere [19]. The accelerometer was attached to the patient’s non-dominant hip with an elastic band, and the patients were instructed to wear the device for seven consecutive days during waking hours [26]. The device was to be removed before water activities and bedtime. Participants providing ≥ 4 days of ≥ 10 hours per day were included in the analysis [26, 27]. Any 90-minute period of consecutive zero counts, with allowance for 2-min intervals of nonzero counts, was categorised as non-wear time [27].

The day was partitioned into absolute and relative time spent in SB, LIPA and MVPA based on vertical axis cut-points of the accelerometers. SB was defined as < 100 counts/min [28], LIPA as 100–2019 counts/min and MVPA as ≥ 2020 counts/min [16]. The relative time of a movement behaviour was calculated based on an isometric log-ratio data transformation, adopting a compositional approach [29]. An isometric log-ratio variable represents, then, the relative importance of one movement behaviour (e.g. MVPA) relative to the geometric average of the other movement behaviours (e.g. SB and LIPA).

### Selection of predictors and potential confounders for SB and MVPA at 6 and 12 months post-surgery

The selection of predictors and potential confounders were based on a revised version of the fear-avoidance model presented in detail in Lotzke et al. [19] and previous studies on the prediction of outcomes of lumbar spine surgery for lumbar degenerative conditions [23, 24, 30]. Moreover, due to limited research on accelerometer-measured SB and MVPA specifically among patients undergoing spine surgery for lumbar degenerative conditions, the selection of potential confounders was also based on research of a broader population [31].

To prevent potential overfitting in the regression models (described below), the number of included potential confounders was limited. As a result, educational attainment and disability measured with the Oswestry Disability Index 2.0 (ODI) [37] were not included as confounders but utilised exclusively for descriptive statistics. While there are known associations between educational attainment and certain surgical outcomes like reoperation rate [31], there is limited evidence linking it to accelerometer-measured MVPA and SB [32]. The ODI was not included as a potential confounder due to its overlapping attributes to back pain intensity measured with a visual analogue scale (VAS, described below). First, the two measures were notably correlated (Spearman’s rho = 0.52) and, second, all items within the ODI are related to back pain, making its content resonate closely with VAS for back pain. Considering the central role of back pain in the fear-avoidance model, VAS for back pain was prioritised over the ODI.

#### Pain-related psychological factors used as predictors

Fear of movement was rated using the Swedish version of the Tampa Scale of Kinesiophobia (TSK) [33]. The TSK ranges from 17 to 68, with higher scores indicating a higher fear of movement. A score of 37 or higher has been considered a high level of fear of movement [17]. Pain catastrophising was measured using the Swedish version of the Pain Catastrophizing Scale (PCS) [34]. The PCS ranges from 0 to 52, with 52 representing the highest level of pain catastrophising. A score of 24 or higher has been identified as clinically relevant [35]. Self-efficacy related to exercise was measured using the Swedish version of the Self-Efficacy for Exercise Scale (SEES) [36]. The SEES ranges from 0 to 90, with a higher score indicating a higher level of self-efficacy for exercise.

#### Potential confounders

Back pain intensity levels over the last week were measured using a 100 mm VAS [37]. Depressive symptoms were assessed using the depression subscale of the Hospital Anxiety and Depression Scale (HADS) [38]. The depression subscale of HADS ranges from 0 (minimum symptoms) to 21 (maximum symptoms). Additional potential confounders were self-reported data on age, gender, body mass index (BMI, kg/m^2^), sick leave at baseline (yes/no), previous back surgery (yes/no), smoking (yes/no), back pain duration (≤ 2 years/>2 years) and participation in the person-centred prehabilitation programme evaluated by Lotzke et al. [25] (yes/no).

### Statistical analysis

#### Descriptive statistics

Descriptive statistics are presented as frequencies, medians, arithmetic means and their respective measures of statistical dispersion. The choice of methods for the descriptive statistics depended on each variable’s data level and distribution. For the absolute and relative time spent in SB, LIPA and MVPA, the geometric mean was calculated across the total population. The geometric mean was chosen instead of the arithmetic mean for the movement behaviour variables as it is less influenced by extreme values in skewed distributions. The descriptive statistics were calculated with SPSS version 27 (IBM Corp., Armonk, NY, USA).

#### Regression analysis

The association between the preoperative pain-related psychological factors and MVPA and SB at 6 and 12 months after surgery was analysed with a linear model regression analysis using the PROC MIXED procedure with an unstructured covariance matrix, performed in SAS version 9.4 (SAS Institute, Cary, NC, USA). This procedure accounts for the correlated nature of within-person repeated measures and uses all available data to estimate model parameters and is particularly suitable for longitudinal data where not all participants have the same number of observations.

A total of six regression models were performed using this procedure. Three models had relative time spent in SB at 6 and 12 months post-surgery as the dependent variable, with each model including one of the predictors separately. The remaining three models had relative time spent in MVPA at 6 and 12 months post-surgery as the dependent variable, with each model also including one of the predictors separately. The confounders were the same for all regression models. The regression models were controlled for the preoperative value of the dependent variable (e.g. the models with relative time spent in MVPA at 6 and 12 months as the dependent variable were controlled for preoperative time spent in MVPA). The regression models’ assumptions were evaluated by checking multicollinearity between independent variables, as well as assessing the linearity between predictors and outcome, normality of variance and homogeneity of variance. A predictor with a p-value ≤ 0.05 was considered statistically significant.

## Results

### Descriptive statistics

The study included 55 men and 63 women with a mean age of 45.7 (SD = 8.3) years (Table 1). On average, the patients reported a back pain intensity on VAS of 58.1 mm (SD = 19.1), a level of disability on ODI of 36.8 (SD = 12.4), a level of fear of movement on TSK of 38.3 (SD = 8.5), and a level of pain catastrophising on PCS of 22.8 (SD = 8.1).

**Table 1:** Socio-demographic and health characteristics for all patients at baseline

	Full sample n = 118	Incomplete accelerometer data at 6-month follow-up (n = 25)	Incomplete accelerometer data at 12-month follow-up (n = 30)
Age, mean (SD)	45.7 (8.3)	44.8 (7.6)	45.1 (8.5)
Sex, frequency (%)			
Women	63 (53.4%)	12 (48.0%)	14 (46.7%)
Men	55 (46.6%)	13 (52.0%)	16 (53.3%)
Body mass index, mean (SD)	26.3 (3.7)	27.5 (3.5)	27.0 (3.7)
Smoking,^*^ frequency (%)	8 (6.8%)	2 (8.0%)	1 (3.3%)
Education,^*^ frequency (%)			
Elementary school	7 (6.0%)	2 (8.0%)	2 (6.7%)
High school	51 (43.6%)	14 (56%)	14 (46.7%)
University or college	42 (35.9%)	5 (20%)	10 (33.3%)
Vocational education	17 (14.5%)	4 (16%)	4 (13.3%)
Sick leave,^†^ frequency (%)	41 (35.3%)	4 (16.0%)	7 (23.3%)
Previous back surgery, frequency (%)	11 (9.3%)	1 (4.0%)	1 (4.0%)
Back pain duration,^*^ n (%)			
≤ 2 years	29 (24.8%)	4 (16.0%)	7 (23.3%)
> 2 years	88 (75.2%)	21 (84.0%)	23 (76.7)
Allocated to prehabilitation	59 (50.0%)	13 (52.0%)	16 (53.3%)
Back pain intensity (VAS), mean (SD)	58.1 (19.1)	66.6 (17.0)	64.0 (17.7)
Oswestry Disability Index, mean (SD)	36.8 (12.4)	37.0 (11.8)	36.2 (13.1)
Depressive symptoms (HADS) mean (SD)	5.4 (3.6)	6.0 (3.3)	5.4 (3.5)
Fear of movement (TSK) mean (SD)	38.2 (8.5)	40.7 (8.1)	39.9 (8.4)
Pain Catastrophizing Scale, mean (SD)	22.8 (8.1)	24.9 (8.8)	24.9 (7.5)
Self-efficacy for Exercise Scale, mean (SD)	61.2 (20.5)	55.2 (24.9)	55 (21.1)

HADS, Hospital Anxiety and Depression Scale; SD, Standard deviation; TSK, Tampa Scale for Kinesiophobia; VAS, Visual analogue scale

^*^ n = 117

^†^ n = 116

Of the total sample, 9.3% had previously undergone surgery for disc herniation. Three patients underwent reoperation within 12 months of the initial surgery. All available data for these individuals were retained in the analysis. Twenty-five patients (21.2%) and 30 patients (25.4%) did not have any accelerometer data at 6 and 12 months, respectively. The characteristics of these subgroups of patients are presented in Table 1. The subgroups were similar for all characteristics, but the patients in the subgroups had a lower rate of sick leave (16.0% and 23.3% in the subgroups compared to 35.3% in the whole sample) and previous lumbar surgery (4.0% in the subgroups compared to 9.3% in the whole sample). Descriptive data for time spent in SB, LIPA and MVPA at baseline, 6 and 12 months, are presented in Table 2. The standard deviation was large at all time points for all movement behaviours, especially for MVPA, indicating a large individual variation.

**Table 2:** Geometric mean (SD) for time spent in SB, LIPA and MVPA at 6 and 12 months, compared to baseline, in minutes/day

	Baseline	6 months	12 months
SB min/day (SD)	541.3 (96.2)	558.8 (83.9)	547.4 (89.6)
LIPA min/day (SD)	267.0 (71.4)	250.2 (69.3)	265.6 (69.6)
MVPA min/day (SD)	20.8 (20.6)	22.8 (20.4)	24.0 (21.4)

LIPA, light intensity physical activity; MVPA, moderate to vigorous intensity physical activity; SB, sedentary behaviour; SD, standard deviation

### Associations between pain-related psychological factors and relative time spent in SB at 6 and 12 months after surgery

Preoperative fear of movement was significantly associated with relative time spent in SB at 6 and 12 months after surgery (β = 0.013, 95% CI = 0.004 to 0.022, p = 0.007, Table 3). This result suggests that patients with a higher preoperative fear of movement spent more relative time in SB at 6 and 12 months compared to those with a lower preoperative fear of movement. Neither preoperative pain catastrophising nor self-efficacy for exercise was found to be significantly associated with relative time spent in SB at 6 and 12 months (Supplementary file 1).

**Table 3:** Regression model for relative time spent in sedentary behaviour at 6 months and 12 months with fear of movement as a predictor

	β	95% CI for β		Standard Error	P
		Lower	Upper		
**Intercept**	0.761	-0.127	1.648	0.447	0.092
**Age**	0.004	-0.005	0.013	0.004	0.377
**Gender**	-0.025	-0.183	0.133	0.079	0.749
**Body mass index**	0.016	-0.003	0.035	0.010	0.101
**Smoking**	-0.153	-0.423	0.116	0.136	0.261
**Sick leave**	-0.029	-0.176	0.117	0.074	0.692
**Previous surgery**	-0.120	-0.358	0.117	0.120	0.317
**Prehabilitation**	0.066	-0.074	0.206	0.071	0.353
**Back pain intensity (VAS)**	-0.001	-0.005	0.003	0.002	0.581
**Depressive symptoms (HADS)**	0.011	-0.010	0.031	0.010	0.296
**Fear of movement (TSK)**	0.013	0.004	0.022	0.005	0.007

CI, Confidence interval; HADS, Hospital Anxiety and Depression Scale; TSK, Tampa Scale for Kinesiophobia; VAS, Visual analogue scale

p ≤ 0.05 indicates statistical significance

### Associations between pain-related psychological factors and relative time spent in MVPA at 6 and 12 months after surgery

Neither preoperative fear of movement, pain catastrophising nor self-efficacy for exercise was significantly associated with relative time spent in MVPA at 6 and 12 months after surgery (Supplementary file 1).

## Discussion

To our knowledge, this is the first study to explore the association between pain-related psychological factors and postoperative SB and MVPA in patients with LBP and degenerative disc disorder, up to 12 months following lumbar fusion surgery. We found that preoperative fear of movement was significantly associated with SB, at 6 and 12 months after surgery. This key finding suggests that patients who enter surgery with preoperative fear of movement have an increased likelihood of poor health after surgery. More specifically, patients with a high level of SB may be more inclined to suffer from the well-documented adverse effects of the “Sedentary Death Syndrome”, including negative cardiovascular, musculoskeletal, metabolic and physiological outcomes [39].

So far, fear of movement has mainly been studied in relation to physical activity rather than to SB. A study by Mancuso et al. [40], involving 260 patients who underwent lumbar spine surgery for degenerative conditions, acknowledged the short- and long-term benefits of physical activity on their spine health and their overall health, but many were deterred from increasing their level of physical activity due to spine-related concerns. More specifically, patients with high levels of *postoperative* fear-avoidance of physical activity and disability were less likely to think walking was a good idea. These results align with our previous results, based on baseline data from the population in the current study, in which higher *preoperative* levels of fear of movement and disability were associated with fewer steps per day before surgery [14]. The current study adds to the previous knowledge in that patients’ preoperative fear of movement was associated with postoperative SB at 6 and 12 months after surgery rather than their level of physical activity.

Our results also demonstrated that pain catastrophising was not significantly associated with either SB or MVPA, 6 and 12 months post-surgery. This finding contrasts with a recent meta-analysis that pointed to studies where pain catastrophising was a significant predictor for disability, pain and health-related quality of life outcomes [23]. The discrepancy could potentially be attributed to variations in the outcome measures used, as no studies within that meta-analysis considered the role of pain catastrophising in relation to postoperative SB and MVPA [23].

Furthermore, self-efficacy for exercise was not significantly associated with either SB or MVPA in the current study. Interestingly, one of our prior studies with the same patient population reported that self-efficacy for exercise was significantly associated with changes in absolute time in MVPA six months after surgery [24]. This finding diverges from our current study, even with the same study sample, illustrating the complex and multifaceted nature of movement behaviour post-surgery. The earlier study was confined to six-month post-operative data and did not account for important confounding factors such as age and BMI, which could explain the contradictory results. Self-efficacy has also been investigated as a potential mediator for postoperative outcomes, as proposed by Woby et al. [18]. For instance, Fors et al. [41] recently demonstrated that changes in self-efficacy mediated the effects of presurgical physiotherapy on disability, back pain intensity and health-related quality of life. However, when we probed whether self-efficacy for exercise mediated the relationship of later changes in MVPA in another study, we found no significant results [42]. Our findings in the current study, when considered alongside previous research [18, 24, 41, 42], prompt further investigation into the role of preoperative self-efficacy. A valuable direction could be to investigate whether preoperative self-efficacy could mediate the effects of presurgical interventions on postoperative SB, in addition to MVPA.

Regarding the generalizability of the results, the patients’ mean age and level of disability and pain are similar to the data in the Swedish spine register [8]. This suggests that the study population comprises a representative sample of the target population regarding these factors. However, it is important to acknowledge that the findings may be less generalizable to the older spectrum of patients undergoing lumbar fusion surgery as well as for patient populations with notably higher or lower levels of disability and pain than those observed in our sample.

### Clinical implications

Given that preoperative fear of movement was significantly associated with postoperative SB, it could be beneficial to integrate a screening procedure for pain-related psychological factors, such as fear of movement, into the preoperative evaluation of patients with LBP and degenerative disc disorder. By early identification of patients at risk for high postsurgical SB, interventions aimed at mitigating preoperative fear of movement could potentially reduce postsurgical SB for these patients.

### Strengths and limitations

The primary strength of the study is that accelerometers were used to measure physical activity, as they yield more objective data than patient-reported outcome measures of physical activity [43]. To our knowledge, this is also one of the first studies of patients undergoing lumbar spine surgery that has included longitudinal accelerometer data up to 12 months after surgery. Nevertheless, hip-worn accelerometers primarily measure walking and running, and may therefore underestimate MVPA for individuals who engage in other types of physical activity [44]. Another strength of the study was that we used the relative time of MVPA and SB as the outcome, thus acknowledging the time spent in all behaviours simultaneously. We also accounted for the correlated nature of within-person repeated measures and missing observations during data analysis.

A limitation of the study is that 25 and 30 patients did not have any accelerometer data at 6 and 12 months, respectively. Although the characteristics of the excluded patients did not deviate notably from those included in the analysis, this limitation could have impacted the robustness of the findings.

Another limitation to consider in our findings relates to their predictive capability. While we found an association between preoperative fear of movement and postoperative movement behaviour, this must not be mistaken for genuine predictive capability. Building a robust predictive model demands a more rigorous research design, encompassing elements such as validation datasets and larger sample sizes [45]. Consequently, while our results provide valuable preliminary insights, they also highlight the need for more comprehensive research approaches.

## Conclusions

Our study demonstrated that preoperative fear of movement was significantly associated with postoperative SB in patients with LBP and degenerative disc disorder undergoing lumbar fusion surgery. This finding underscores the potential benefits of providing preoperative screening of pain-related psychological factors, including fear of movement, to identify patients who are suitable for preoperative interventions aimed at a healthier postoperative movement behaviour and improved health.

### Electronic supplementary material

Below is the link to the electronic supplementary material.

Supplementary Material 1

## Data Availability

The datasets used for the current study are available from the corresponding author upon reasonable request.

## List of abbreviations

BMI: body mass index
HADS: Hospital Anxiety and Depression scale
LIPA: light intensity physical activity
MVPA: moderate to vigorous physical activity
ODI: Oswestry Disability Index 2.0
PCS: Pain Catastrophizing Scale
SB: sedentary behaviour
SEES: Self-Efficacy for Exercise Scale
TSK: Tampa Scale for Kinesiophobia
VAS: Visual Analogue Scale
WHO: World Health Organization
