# Supplementary material for: Fear of movement was associated with sedentary behaviour 12 months after lumbar fusion surgery in patients with low back pain and degenerative disc disorder
Source: BMC Musculoskelet Disord. 2023 Nov 10;24:874. doi: 10.1186/s12891-023-06980-z (PMC10636920; doi:10.1186/s12891-023-06980-z)
Supplement: Supplementary file 1 — Supplementary Material 1 [file 12891_2023_6980_MOESM1_ESM.docx]

**Table S1.** Regression model for relative time spent in sedentary behaviour at 6 months and 12 months with pain catastrophizing as a predictor

|  | **β** | **95% CI for β** | | **Standard Error** | **P** |
| --- | --- | --- | --- | --- | --- |
|  |  | **Lower** | **Upper** |  |  |
| **Intercept** | 1.361 | 0.497 | 2.226 | 0.436 | 0.002 |
| **Age** | 0.000 | -0.008 | 0.009 | 0.004 | 0.931 |
| **Gender** | 0.041 | -0.110 | 0.191 | 0.076 | 0.593 |
| **Body mass index** | 0.017 | -0.003 | 0.037 | 0.010 | 0.092 |
| **Smoking** | -0.175 | -0.460 | 0.110 | 0.144 | 0.226 |
| **Sick leave** | -0.024 | -0.173 | 0.125 | 0.075 | 0.752 |
| **Previous surgery** | -0.146 | -0.387 | 0.096 | 0.122 | 0.235 |
| **Prehabilitation** | 0.057 | -0.086 | 0.199 | 0.072 | 0.434 |
| **Back pain intensity (VAS)** | 0.000 | -0.004 | 0.004 | 0.002 | 0.962 |
| **Depressive symptoms (HADS)** | 0.020 | -0.002 | 0.041 | 0.011 | 0.080 |
| **Pain catastrophising (PCS)** | -0.004 | -0.014 | 0.007 | 0.005 | 0.489 |

CI, Confidence interval; HADS, Hospital Anxiety and Depression Scale; PCS, Pain Catastrophizing Scale; VAS, Visual analogue Scale

p ≤ 0.05 indicates statistical significance

**Table S2.** Regression model for relative time spent in sedentary behaviour at 6 months and 12 months with self-efficacy for exercise as a predictor

|  | **β** | **95% CI for β** | | **Standard Error** | **P** |
| --- | --- | --- | --- | --- | --- |
|  |  | **Lower** | **Upper** |  |  |
| **Intercept** | 1.269 | 0.404 | 2.133 | 0.436 | 0.004 |
| **Age** | 0.001 | -0.008 | 0.010 | 0.004 | 0.848 |
| **Gender** | 0.042 | -0.110 | 0.193 | 0.076 | 0.587 |
| **Body mass index** | 0.016 | -0.004 | 0.036 | 0.010 | 0.107 |
| **Smoking** | -0.150 | -0.426 | 0.127 | 0.140 | 0.286 |
| **Sick leave** | -0.026 | -0.178 | 0.126 | 0.077 | 0.733 |
| **Previous surgery** | -0.147 | -0.391 | 0.098 | 0.123 | 0.236 |
| **Prehabilitation** | 0.056 | -0.090 | 0.201 | 0.073 | 0.450 |
| **Back pain intensity (VAS)** | 0.000 | -0.004 | 0.004 | 0.002 | 0.999 |
| **Depressive symptoms (HADS)** | 0.016 | -0.004 | 0.037 | 0.010 | 0.106 |
| **Self-efficacy for exercise (SEES)** | 0.001 | -0.022 | 0.025 | 0.012 | 0.908 |

CI, Confidence interval; HADS, Hospital Anxiety and Depression Scale; SEES, Self-efficacy for Exercise Scale; VAS, Visual analogue scale
p ≤ 0.05 indicates statistical significance

**Table S3.** Regression model for relative time spent in moderate to vigorous physical activity at 6 months and 12 months with fear of movement as a predictor

|  | **β** | **95% CI for β** | | **Standard Error** | **P** |
| --- | --- | --- | --- | --- | --- |
|  |  | **Lower** | **Upper** |  |  |
| **Intercept** | -1.411 | -2.831 | 0.009 | 0.716 | 0.051 |
| **Age** | -0.006 | -0.020 | 0.008 | 0.007 | 0.381 |
| **Gender** | 0.223 | -0.028 | 0.475 | 0.127 | 0.081 |
| **Body mass index** | -0.029 | -0.060 | 0.002 | 0.016 | 0.065 |
| **Smoking** | 0.583 | 0.150 | 1.016 | 0.218 | 0.009 |
| **Sick leave** | 0.124 | -0.111 | 0.359 | 0.118 | 0.298 |
| **Previous surgery** | 0.161 | -0.220 | 0.541 | 0.192 | 0.405 |
| **Prehabilitation** | -0.162 | -0.386 | 0.062 | 0.113 | 0.156 |
| **Back pain intensity (VAS)** | 0.000 | -0.007 | 0.006 | 0.003 | 0.904 |
| **Depressive symptoms (HADS)** | -0.011 | -0.043 | 0.021 | 0.016 | 0.499 |
| **Fear of movement (TSK)** | -0.014 | -0.029 | 0.001 | 0.007 | 0.063 |

CI, Confidence interval; HADS, Hospital Anxiety and Depression Scale; TSK, Tampa Scale for Kinesiophobia; VAS, Visual analogue scale
p ≤ 0.05 indicates statistical significance

**Table S4.** Regression model for relative time spent in moderate to vigorous physical activity at 6 months and 12 months with pain catastrophizing as a predictor.

|  | **β** | **95% CI for β** | | **Standard Error** | **P** |
| --- | --- | --- | --- | --- | --- |
|  |  | **Lower** | **Upper** |  |  |
| **Intercept** | -2.014 | -3.379 | -0.649 | 0.688 | 0.004 |
| **Age** | -0.003 | -0.017 | 0.011 | 0.007 | 0.692 |
| **Gender** | 0.153 | -0.085 | 0.391 | 0.120 | 0.205 |
| **Body mass index** | -0.030 | -0.061 | 0.001 | 0.016 | 0.060 |
| **Smoking** | 0.596 | 0.144 | 1.048 | 0.228 | 0.010 |
| **Sick leave** | 0.114 | -0.122 | 0.350 | 0.119 | 0.340 |
| **Previous surgery** | 0.180 | -0.203 | 0.563 | 0.193 | 0.354 |
| **Prehabilitation** | -0.149 | -0.375 | 0.077 | 0.114 | 0.193 |
| **Back pain intensity (VAS)** | -0.001 | -0.008 | 0.005 | 0.003 | 0.638 |
| **Depressive symptoms (HADS)** | -0.012 | -0.055 | 0.015 | 0.018 | 0.256 |
| **Pain catastrophizing (PCS)** | 0.003 | -0.013 | 0.019 | 0.008 | 0.740 |

CI, Confidence interval; HADS, Hospital Anxiety and Depression Scale; PCS, Pain Catastrophizing Scale; VAS, Visual analogue Scale
p ≤ 0.05 indicates statistical significance

**Table S5.** Regression model for relative time spent in moderate to vigorous physical activity at 6 months and 12 months with self-efficacy for exercise as a predictor

|  | **β** | **95% CI for β** | | **Standard Error** | **P** |
| --- | --- | --- | --- | --- | --- |
|  |  | **Lower** | **Upper** |  |  |
| **Intercept** | -2.189 | -3.541 | -0.836 | 0.682 | 0.002 |
| **Age** | -0.003 | -0.016 | 0.011 | 0.007 | 0.717 |
| **Gender** | 0.169 | -0.068 | 0.406 | 0.120 | 0.161 |
| **Body mass index** | -0.028 | -0.059 | 0.003 | 0.016 | 0.080 |
| **Smoking** | 0.542 | 0.108 | 0.977 | 0.219 | 0.015 |
| **Sick leave** | 0.089 | -0.149 | 0.326 | 0.120 | 0.461 |
| **Previous surgery** | 0.141 | -0.243 | 0.525 | 0.194 | 0.467 |
| **Back pain intensity (VAS)** | -0.001 | -0.007 | 0.005 | 0.003 | 0.813 |
| **Depressive symptoms (HADS)** | -0.015 | -0.047 | 0.016 | 0.016 | 0.341 |
| **Prehabilitation** | -0.176 | -0.404 | 0.052 | 0.115 | 0.128 |
| **Self-efficacy for exercise (SEES)** | 0.025 | -0.012 | 0.062 | 0.018 | 0.177 |

CI, Confidence interval; HADS, Hospital Anxiety and Depression Scale; SEES, Self-efficacy for Exercise Scale; VAS, Visual analogue scale
p ≤ 0.05 indicates statistical significance
